# How do hypoglycaemias affect the everyday life of people with diabetes and are they able to treat them adequately?

**DOI:** 10.1111/dme.70121

**Published:** 2025-08-16

**Authors:** Nicolle Müller, Christiane Kellner, Sebastian Schmidt, Nadine Kuniß, Gunter Wolf, Christof Kloos

**Affiliations:** ^1^ Department for Internal Medicine III Jena University Hospital Jena Germany; ^2^ Outpatient Healthcare Center MED:ON MVZ Erfurt Germany

**Keywords:** fear of hypoglycaemia, hypoglycaemia treatment, impaired hypoglycaemia awareness

## Abstract

**Aims:**

To investigate whether people with type 1 and type 2 diabetes respond adequately to hypoglycaemia symptoms after participating in a treatment and teaching programme. Additionally, it explored how hypoglycaemia impacts patients' everyday life and the differences between individuals with and without impaired awareness of hypoglycaemia (IAH).

**Methods:**

This cross‐sectional study included 340 adult participant with type 1 (*n* = 156) or type 2 diabetes (*n* = 184) undergoing insulin therapy at the University Hospital Jena. Participants completed validated questionnaires and participated in structured interviews about hypoglycaemia. Awareness of hypoglycaemia was measured using the Gold Score, and diabetes distress with the PAID Scale (PAID Score 0–100, the higher the score the higher the distress).

**Results:**

23.8% of the participants treated hypoglycaemia adequately (type 1 diabetes 27.6% vs. type 2 diabetes 24.7%; *p* = 0.606). Significantly more people without IAH‐treated hypoglycaemia adequately in type 2 (27.9% vs. 10.3%; *p* = 0.047) but not in type 1 diabetes (28.6% vs. 23.1%; *p* = 0.568). Hypoglycaemia altered daily routines for 26.2% of participants, particularly those with type 1 diabetes (type 1 diabetes 37.3% vs. type 2 diabetes 20.1%; *p* = 0.001). People with type 1 and type 2 diabetes reporting changes in daily routines had higher diabetes distress scores (PAID: 22.3 ± 16.0 vs. 13.8 ± 13.5; *p* < 0.001). Fear of hypoglycaemia was associated with higher HbA_1c_ values due to people accepting elevated blood glucose levels.

**Conclusions:**

Despite education programmes, the majority of participants do not treat hypoglycaemia adequately. Hypoglycaemic events significantly impact daily life and are associated with increased diabetes‐related distress, especially in those with IAH.

**What's new?**

**What is already known?**

- Hypoglycaemia is a significant complication in diabetes management and affects people' quality of life and increases diabetes‐related distress.
- Patient education programmes improve hypoglycaemia management and awareness.

**What this study has found?**

- Only 23.8% of the participants treat hypoglycaemia adequately despite education programmes.
- Hypoglycaemia is significantly associated with altered daily routines.
- Fear of hypoglycaemia is associated with higher HbA_1c_ values.

**What are the implications of the study?**

- Improved patient education is essential to optimise hypoglycaemia management; continuous glucose monitoring can facilitate this.
- Addressing fear of hypoglycaemia is crucial to achieve better glycaemic control and quality of life of individuals with diabetes.

## 1 INTRODUCTION

Despite new drugs and technologies in diabetes therapy, hypoglycaemia remains a significant acute complication in people using insulin and insulin‐releasing therapy.^1^ Furthermore, hypoglycaemia often inhibits achieving good glycaemic control.^2^, ^3^ If glucose levels drop below 3.9 mmol/L, counterregulatory mechanisms are activated and adrenocortical hormones are released, causing typical symptoms of hypoglycaemia such as trembling, hunger, and nervousness. If the glucose level continues to decrease and drops below 3 mmol/L, glucose deficiency in the central nervous system finally leads to cognitive impairment and even to seizures or coma.^4^, ^5^ These symptoms, the sometimes prolonged recovery from hypoglycaemic episodes, and the feeling of helplessness during severe hypoglycaemia negatively impact well‐being and may lead to fear of hypoglycaemia.^6^

A key element of structured treatment and teaching programmes is to prepare people with diabetes and insulin therapy to prevent hypoglycaemia or to react appropriately if symptoms of hypoglycaemia occur. As part of these training programmes, people learn which foods rapidly increase blood glucose and are suited for treating hypoglycaemia, as well as how to prevent hypoglycaemia during exercise.^7^ The effectiveness of participation in a treatment and teaching programme to reduce severe hypoglycaemia has been demonstrated in some studies for both type 1 and type 2 diabetes mellitus.^8^, ^9^, ^10^, ^11^ participants with impaired awareness of hypoglycaemia (IAH) represent a particular challenge in terms of care. Their risk of severe hypoglycaemia is markedly increased, associated with increased but also reduced fear of hypoglycaemia, and negatively affects their quality of life.^6^, ^12^, ^13^

In the present study, we investigated if people with type 1 diabetes and type 2 diabetes, who have participated in a treatment and teaching programme in the past, adequately respond to symptoms of hypoglycaemia and what impact the occurrence of hypoglycaemia had on their everyday life. Furthermore, we examined for differences in people with and without IAH.

## 2 PARTICIPANTS AND METHODS

### 2.1 Patients

This cross‐sectional study was performed at the outpatient Department of Endocrinology and Metabolic Diseases at the University Hospital Jena. All adult participants presenting between August 2021 and February 2022 were included if type 1 diabetes or type 2 diabetes with insulin therapy was present, if they were able to understand and speak the German language, and were willing to fill out questionnaires. Exclusion criteria were newly diagnosed diabetes and illnesses impairing the ability to complete the questionnaire (e.g., dementia). All participants have taken part in a treatment and teaching programme at least once.

After written consent to participate, all participants received several validated questionnaires and were interviewed specifically about hypoglycaemia.

The study was approved by the local ethics committee (number 2021‐2312‐Bef).

### 2.2 Assessments

The participants participated in a structured interview and were asked what procedure they would pursue in case of a hypoglycaemic event. The answers were appraised and regarded as adequate treatment of hypoglycaemia if the measures matched the procedures of the education programme consisting of promptly eating or drinking 15–25 g of rapidly absorbable carbohydrates and measuring blood glucose immediately afterwards. Furthermore, participants were asked what measures they take to prevent hypoglycaemia and if and in what means their everyday life was affected by the hypoglycaemia.

Awareness of hypoglycaemia was measured using the Gold Score, which consists of the question: ‘Do you know when your hypos are commencing?’ The answer is rated on a Lickert scale from 1 ‘always aware’ to 7 ‘never aware’ A score of 4 or more indicates IAH.^14^

Subjective well‐being was assessed using the WHO‐Five Well‐Being Index (WHO5). Participants were asked to indicate for each of the five statements how they felt over the past 2 weeks. Each of the 5 items is scored on a Likert scale from 0 (none of the time) to 5 (all of the time). The score ranges from 0 to 25, a higher score indicating higher well‐being.^15^

Diabetes distress was assessed using the Problem Areas In Diabetes (PAID) Scale. Twenty items focus on different problem areas of diabetes on a 5‐point Likert scale from 0 (not a problem) to 4 (serious problem). All 20 items are added and multiplied by 1.25 to give a score of 0–100. Higher scores indicate more diabetes‐related distress (cut‐off ≥40 indicates high distress).^16^

Social status was obtained from all participants using a validated questionnaire. Social status, ranging from a minimum of 3 to a maximum of 21 points, was composed of education, highest professional position, and household net income.^17^

### 2.3 Parameters

Laboratory and clinical data were drawn from the digital patient record EMIL®^18^ and were collected on the day of the survey.

Non‐severe hypoglycaemia was defined as typical symptoms that disappear quickly after the intake of carbohydrate or plasma glucose ≤3.9 mmol/L without typical symptoms. Severe hypoglycaemia was defined as requiring the assistance of another person.^19^

### 2.4 Statistical analyses

Statistical analyses were performed with SPSS 27 (IBM Corporation, Armonk, NY, USA). Normally distributed values were registered as mean ± SD and non‐normally distributed values as median and range. Comparisons were evaluated with the Chi‐squared test and incidences <6 with the exact test of Fischer. Student's *t*‐test and Wilcoxon tests were used to compare the means. Significance was defined at the 0.05 level.

## 3 RESULTS

A total of 340 insulin‐treated people were included in the study, 156 with type 1 diabetes (35.3% with CSII and 64.7% with ICT) and 184 with type 2 diabetes. The characteristics of the included individuals are shown in Table 1. About 16.7% of participants with type 1 diabetes and 17.4% of participants with type 2 diabetes have impaired hypoglycaemia awareness (IAH). The characteristics of these participants are given in Table 2.

**TABLE 1:** Characteristics of all patients.

Parameter	T1DM (*n* = 156)	T2DM (*n* = 184)
Women *n* (%)	66 (42.3)	67 (36.4)
Age (years)	56.1 ± 17.3	70.3 ± 11.6
Diabetes duration (years)	25.4 ± 14.0	20.8 ± 9.3
BMI (kg/m^2^)	28.1 ± 5.3	33.6 ± 7.6
Blood pressure sys (mmHg)	143.2 ± 17.3	146.2 ± 19.5
Blood pressure dias (mmHg)	82.7 ± 11.0	81.8 ± 14.0
HbA_1c_ (%)	8.2 ± 1.0	8.0 ± 1.0
HbA_1c_ (mmol/mol)	66.1 ± 12.6	63.9 ± 12.6
Non‐severe hypoglycaemia per month	7.4 ± 8.6	0.9 ± 3.2
Severe hypoglycaemia per year	0.05 ± 0.29	0.06 ± 0.54
Social status	12.2 ± 4.3	11.7 ± 3.8
Smoking—yes *n* (%)	32 (20.5)	30 (16.3)
CGM usage *n* (%)	106 (67.9)	36 (19.6)
GFR (CKD‐EPI) (mL/min/1.73 m^2^)	84.6 ± 25.8	60.4 ± 24.7
Kreatinin (μmol/L)	88.5 ± 51.5	123.0 ± 100.2

**TABLE 2:** Characteristics of patients with IAH.

Parameter	T1DM (*n* = 26)	T2DM (*n* = 32)
Women *n* (%)	6 (23.1)	14 (43.8)
Age (years)	60.5 ± 16.2	69.4 ± 11.5
Diabetes duration (years)	26.5 ± 15.4	15.1 ± 6.9
BMI (kg/m^2^)	28.9 ± 4.3	35.5 ± 10.6
Blood pressure sys (mmHg)	142.0 ± 19.3	146.8 ± 24.2
Blood pressure dias (mmHg)	78.8 ± 10.7	81.9 ± 12.4
HbA_1c_ (%)	8.0 ± 1.0	8.0 ± 1.3
HbA_1c_ (mmol/mol)	63.9 ± 12.6	63.9 ± 9.3
Non‐severe hypoglycaemia per month	7.7 ± 9.4	0.6 ± 1.7
Severe hypoglycaemia per year	0.11 ± 0.59	0.06 ± 0.25
Smoking—yes *n* (%)	5 (19.2)	5 (15.6)
Social status	11.5 ± 4.5	10.8 ± 1.3
CGM usage *n* (%)	21 (80.8)	8 (25.0)
GFR (CKD‐EPI) (mL/min/1.73 m^2^)	79.6 ± 30.0	59.3 ± 24.6
Kreatinin (μmol/L)	107.3 ± 88.7	115.3 ± 60.4

### 3.1 Treatment of hypoglycaemia

Only 23.8% of the participants treat their hypoglycaemia adequately (type 1 diabetes 27.6% vs. type 2 diabetes 24.7%; *p* = 0.606). 57.8% of type 1 and 54.1% of type 2 diabetes first measure the blood glucose in situations with clinical signs of hypoglycaemia before ingesting carbohydrates. 52.6% of the participants without the usage of continuous glucose monitoring (CGM) act accordingly.

The frequency of adequate treatment of people with type 1 diabetes without and with IAH (28.6% vs. 23.1%; *p* = 0.568) does not differ. In participants with type 2 diabetes, more participants without IAH react adequately than with IAH (27.9% vs. 10.3%; *p* = 0.047). 48.1% of the participants with type 1 diabetes take 15–25 g of carbohydrates in case of symptoms of hypoglycaemia. There was no difference between participants with CGM and without CGM use (47.3% vs. 50.0%; *p* = 0.924). 43.0% of the participants with type 1 diabetes take more than 25 g of carbohydrates. Among participants with type 2 diabetes, 47.1% take 15–25 g of carbohydrates. There also was no difference between participants with and without CGM (56.7% vs. 44.4%; *p* = 0.387). 27.5% of the participants with type 2 diabetes take more than 25 g. Significantly more participants with type 1 diabetes and IAH take more than 25 g of carbohydrates in case of symptoms of hypoglycaemia than participants without IAH (68.4% vs. 43.3%; *p* = 0.049). Numerically, this is also the case for participants with type 2 diabetes and IAH but lacks significance (42.1% vs. 35.7%; *p* = 0.608).

Participants not treating their hypoglycaemia adequately suffer from significantly more severe hypoglycaemia in the last 12 months (0.02 vs. 0.0; *p* = 0.045).

### 3.2 Prevention of hypoglycaemia

After a hypoglycaemic episode, 64.7% of participants stated they would make preparations to prevent another hypoglycaemic episode in this situation. This stated significantly more participants with type 1 than with type 2 diabetes (82.4% vs. 59.5%; *p* < 0.001) independently from having IAH (type 1 diabetes without IAH 80.3% vs. with IAH 92.3%; *p* = 0.170; type 2 diabetes without IAH 57.4% vs. with IAH 69.0%; *p* = 0.250). This stated also significantly more participants with type 1 diabetes and CGM use than without CGM (89.5% vs. 66.7%; *p* = 0.001). This was also the case in people with type 2 diabetes (70.6% vs. 56.5%; *p* = 0.097).

### 3.3 Influence of hypoglycaemia on people' everyday lives

About 26.2% of the participants reported that the occurrence of hypoglycaemia changed their everyday life. This is significantly more often the case in people with type 1 compared with type 2 diabetes (37.3% vs. 20.1%; *p* = 0.001). IAH was similarly often present in these participants in type 1 (without 37.0% vs. 38.5% with IAH; *p* = 0.889) and type 2 diabetes (20.2% without vs. 20.0% with IAH; *p* = 0.985). Also, the use of CGM shows no difference in type 1 (with CGM 39.0% vs. without CGM 33.3%; *p* = 0.311) and type 2 diabetes (with CGM 23.5% vs. without CGM 19.2%; *p* = 0.366).

**FIGURE 1:**
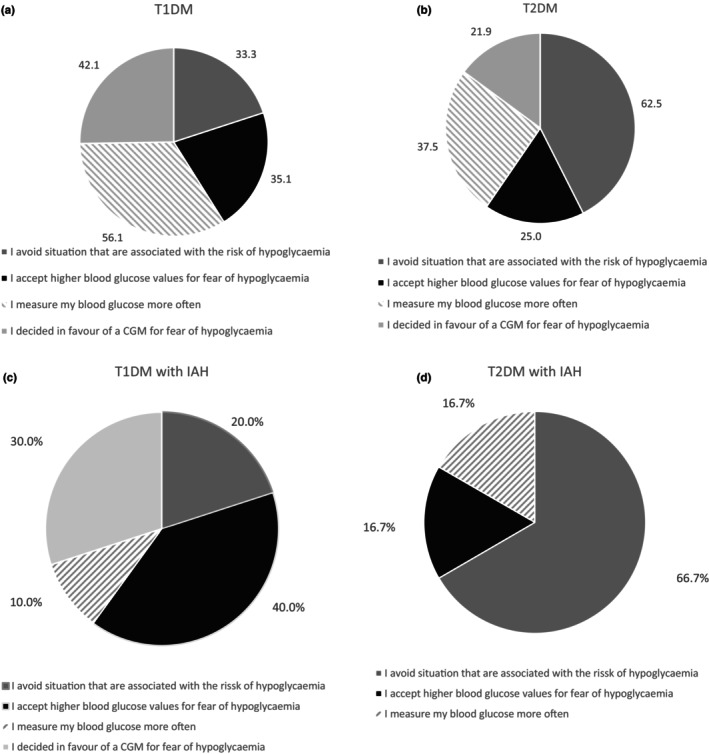
Changes of everyday life due to hypoglycaemia in (a) type 1 diabetes, (b) type 2 diabetes, (c) type 1 diabetes with IAH, (d) type 2 diabetes with IAH (multiple applications possible).

People who have changed their daily routine due to hypoglycaemia have a significantly greater diabetes distress (PAID score 22.3 ± 16.0 vs. 13.8 ± 13.5; *p* < 0.001). This is the case for either participants with type 1 (22.7 ± 15.1 vs. 13.3 ± 13.7; *p* < 0.001) and type 2 diabetes (21.5 ± 17.8 vs. 14.1 ± 13.4; *p* = 0.036). The well‐being index is comparable in participants with type 1 (15.5 ± 4.8 vs. 16.9 ± 4.2; *p* = 0.064) and type 2 diabetes (16.0 ± 5.0 vs. 15.5 ± 5.6; *p* = 0.633). Participants with type 1 diabetes stated predominantly that they measure the blood glucose more frequently and have opted for a CGM due to fear of hypoglycaemia. Participants with type 2 diabetes preponderantly stated to avoid situations associated with hypoglycaemia and furthermore to measure the blood glucose more frequently (Figure 1a,b). The majority of participants with type 1 diabetes and IAH reported accepting higher blood glucose values and also having opted for a CGM. Concerning people with type 2 diabetes and IAH, the most prevalent measures taken were to avoid situations associated with hypoglycaemia, to measure the blood glucose more frequently, and to accept higher blood glucose values (Figure 1c,d). Participants reporting accepting higher blood glucose levels due to fear of hypoglycaemia had a significantly higher HbA_1c_ value (all participants: 8.6% ± 1.0 vs. 8.0% ± 1.0; *p* = 0.002; type 1 diabetes 8.6% ± 1.2 vs. 8.2% ± 1.0; *p* = 0.053; type 2 diabetes 8.7% ± 0.6 ± 7.9% ± 1.0; *p* = 0.042).

More participants with type 1 diabetes without CGM usage stated they avoided situations associated with hypoglycaemia (without CGM 43.8% vs. with CGM 21.1%; *p* = 0.231) but the difference was not significant. In the acceptance of higher blood glucose values (without CGM 37.5% vs. with CGM 34.1%; *p* = 0.522) and the more frequent measurement of blood glucose, there was no difference (without CGM 56.3% vs. with CGM 56.1%; *p* = 0.641). Participants with type 2 diabetes without CGM significantly more often stated they avoided situations associated with hypoglycaemia (without CGM 75.0% vs. with CGM 25.0%; *p* = 0.018). A numerical but non‐significant difference was found in the acceptance of higher blood glucose values (without CGM 29.2% vs. with CGM 12.5%; *p* = 0.333) and the more frequent measurement of blood glucose (without CGM 29.2% vs. with CGM 62.5%; *p* = 0.104).

## 4 DISCUSSION

The purpose of this study was to analyse how individuals with diabetes manage hypoglycaemic situations and how hypoglycaemia can affect everyday life. Despite participating in a diabetes treatment and teaching programme which includes specific training to handle situations with hypoglycaemia, only 23.8% of the participants manage the therapy of hypoglycaemia adequately. The majority of the participants measure blood glucose first before ingesting carbohydrates, and almost half of them ingest more than 25 g of carbohydrates. A pilot study by Sommerfield et al. also shows that only 40% of the respondents treat their hypoglycaemia as recommended. In contrast to our investigation, though, most participants undertreated hypoglycaemia.^20^ The inadequate treatment of hypoglycaemia may be due to a lack of persistent knowledge despite former training. The understanding of training content is usually only checked during the training period by means of questions or practical training tasks. There are usually no further checks in everyday clinical practice. Furthermore, people may purposely deviate from recommendations based on personal experience, particularly in cases of long‐term illness. Increased carbohydrate intake may, for example, be attributable to physical activity or a reduced frequency of blood glucose monitoring due to a high level of confidence in symptom interpretation. However, these aspects were not assessed during the participant interviews.

A previous study showed that a significant proportion of participants were unable to identify hypoglycaemia and only 24% correctly treated hypoglycaemia.^21^ As a consequence, a lack of knowledge can increase the risk of severe hypoglycaemia with coma, especially in participants with IAH.

Hypoglycaemia affects the daily life of the individuals concerned and drove some to change their lives. This affects particularly people with IAH. In particular, participants reported measuring blood glucose values more frequently and avoiding situations possibly associated with a higher risk of hypoglycaemia. This impact measurably increased diabetes‐related distress. In a large survey by Chatwin et al., hypoglycaemia was found to have a negative impact on people' overall quality of life, especially in the domains of activity, sleep, emotional well‐being, spontaneity, and independence.^6^

The more often hypoglycaemic events occur, the more likely is an association with fear of hypoglycaemia that can impair optimal blood glucose control.^22^, ^23^ In our study, some participants reported accepting higher blood glucose levels because of the fear of hypoglycaemia, particularly those with IAH. This results in inadequate metabolic control with higher HbA_1c_ values and, in the long run, a higher risk of long‐term complications.

CGM can reduce the fear of hypoglycaemia and improve quality of life in participants with type 1 diabetes without and with IAH^24^, ^25^ whereas in participants with type 2 diabetes the evidence is not clear, probably due to the lower frequency of hypoglycaemia in these participants.^26^ In our study, an equal percentage of participants with and without CGM use reported that hypoglycaemia affected their everyday lives. Follow‐up education or targeted intervention programmes represent valuable approaches to improving participants care.^8^, ^9^, ^10^, ^11^ Patient‐reported outcome measures (PROMs) and participant‐reported experience measures (PREMs) can facilitate the identification of individual challenges and support the early detection of participants increased risk.

It is not clear whether the people' behavioural changes such as avoiding situations with a risk of hypoglycaemia or controlling blood glucose more frequently actually reduce the frequency of hypoglycaemia. Especially as the frequency of hypoglycaemia depends on various factors, such as age, HbA_1c_, and kidney function.^27^, ^28^ However, this was not the objective of the present study and was therefore not analysed. It is also plausible that factors beyond hypoglycaemia awareness and the use of CGM systems may influence the extent to which hypoglycaemia affects daily life. These factors may include age, gender, duration of illness, or socioeconomic status, and should be examined in future research. Such findings could result in the development of more individualised training and support programmes.

The study has some limitations that restrict the generalisability of the data. The first limitation is that the study was conducted in a single centre at a university hospital. The participants of this centre may represent a negative selection with more problems in the treatment of their diabetes than individuals at other levels of care. Furthermore, the results are based on self‐reported recollections of actions taken by participants in the event of hypoglycaemic symptoms, as well as the types and quantities of carbohydrates consumed. The adequate treatment of mild hypoglycaemia was defined as first eating or drinking 15–25 g of rapidly absorbable carbohydrates, then measuring blood glucose as recommended in the treatment and teaching programmes and guidelines.^29^, ^30^ On the other hand, the amount of carbohydrate required to treat an episode of mild hypoglycaemia differs individually and varies according to the blood glucose value, the circumstances of the hypoglycaemia and, above all, the participants' experience with the appropriate amount to treat their hypoglycaemia.

## 5 CONCLUSION

Hypoglycaemia remains to be a treatment‐limiting event in the care of individuals with type 1 and type 2 diabetes. It impacts participants' everyday lives and leads to behavioural changes, resulting in greater diabetes‐related distress and suboptimal blood glucose control, especially due to fear of hypoglycaemia. Furthermore, only a quarter of the participants treat their hypoglycaemia adequately. For some individuals, particularly those with type 1 diabetes and those with IAH, CGM is an appropriate way to reduce the frequency of hypoglycaemia and the stress it causes. However, participant education and training, awareness of hypoglycaemic symptoms, adequate treatment, and anticipatory self‐management of insulin therapy remain the cornerstones for all people.

## AUTHOR CONTRIBUTIONS

NM researched data and drafted the report; MF researched data; CK, SS, NK, GW and CK reviewed/edited the manuscript and contributed to the discussion.

## FUNDING INFORMATION

The study was supported by funds provided by the home institutions of the authors.

## CONFLICT OF INTEREST STATEMENT

There are no potential conflicts of interest relevant to this study.

## STATEMENT OF HUMAN AND ANIMAL RIGHTS

All procedures followed were in accordance with the ethical standards of the committee on human experimentation of the study institutions and German national standards as well as with the Helsinki Declaration of 1975, as revised in 2008 (5).

## GUARANTOR STATEMENT

Nicolle Müller is the guarantor of this work and had full access to all study data and computations and takes responsibility for the integrity of the data and accuracy of data analyses.

## Data Availability

The data that support the findings of this study are available on request from the corresponding author. The data are not publicly available due to privacy or ethical restrictions.

## References

[1] Roth M , Lehmann T , Kloos C , et al. Metabolic control, diabetic complications and drug therapy in a cohort of patients with type 1 and type 2 diabetes in secondary and tertiary care between 2004 and 2019. Int J Environ Res Public Health. 2023;20:640‐645. 10.3390/ijerph20032631 PMC9916122 36768000

[2] Inzucchi SE , Bergenstal RM , Buse JB , et al. Management of hyperglycemia in type 2 diabetes: a patient‐centered approach: position statement of the American Diabetes Association (ADA) and the European Association for the Study of diabetes (EASD). Diabetes Care. 2012;35:1364‐1379. 22517736 10.2337/dc12-0413 PMC3357214

[3] Lipska KJ , Warton EM , Huang ES , et al. HbA1c and risk of severe hypoglycemia in type 2 diabetes: the diabetes and aging study. Diabetes Care. 2013;36:3535‐3542. 23900589 10.2337/dc13-0610 PMC3816866

[4] de Galan BE , McCrimmon RJ , Ibberson M , et al. Reducing the burden of hypoglycaemia in people with diabetes through increased understanding: design of the Hypoglycaemia REdefining SOLutions for better liVEs (hypo‐RESOLVE) project. Diabet Med. 2020;37:1066‐1073. 31970814 10.1111/dme.14240 PMC7317819

[5] Davis RE , Morrissey M , Peters JR , Wittrup‐Jensen K , Kennedy‐Martin T , Currie CJ . Impact of hypoglycaemia on quality of life and productivity in type 1 and type 2 diabetes. Curr Med Res Opin. 2005;21:1477‐1483. 16197667 10.1185/030079905X61929

[6] Chatwin H , Broadley M , Hendrieckx C , et al. The impact of hypoglycaemia on quality of life among adults with type 1 diabetes: results from “YourSAY: Hypoglycaemia”. J Diabetes Complicat. 2023;37:108232. 10.1016/j.jdiacomp.2022.108232 35927177

[7] Beck J , Greenwood DA , Blanton L , et al. 2017 National Standards for diabetes self‐management education and support. Diabetes Educ. 2017;43:449‐464. 28753378 10.1177/0145721717722968

[8] Kloos C , Burghardt K , Muller UA , et al. Reduction of severe hypoglycaemia in people with type 2 diabetes after a structured inpatient intervention. Exp Clin Endocrinol Diabetes. 2021;129:587‐592. 31487750 10.1055/a-0983-1559

[9] Muller N , Kloos C , Samann A , Wolf G , Muller UA . Evaluation of a treatment and teaching refresher programme for the optimization of intensified insulin therapy in type 1 diabetes. Patient Educ Couns. 2013;93:108‐113. 23747089 10.1016/j.pec.2013.05.008

[10] Elliott J , Jacques RM , Kruger J , et al. Substantial reductions in the number of diabetic ketoacidosis and severe hypoglycaemia episodes requiring emergency treatment lead to reduced costs after structured education in adults with type 1 diabetes. Diabet Med. 2014;31:847‐853. 24654672 10.1111/dme.12441 PMC4264891

[11] Sämann A , Mühlauser I , Bender R , Kloos C , Müller UA . Glycaemic control and severe hypoglycaemia following training in flexible, intensive insulin therapy to enable dietary freedom in people with type 1 diabetes: a prospective impmenetation study. Diabetologia. 2005;45:1965‐1970. 10.1007/s00125-005-1905-1 16132954

[12] Hatle H , Bjorgaas MR , Ro TB , Olsen SE , Asvold BO . Fear of hypoglycaemia and its relation to hypoglycaemia awareness and symptom intensity in type 1 diabetes. Diabetes Res Clin Pract. 2018;137:213‐220. 29407272 10.1016/j.diabres.2018.01.014

[13] Farrell CM , McCrimmon RJ . Clinical approaches to treat impaired awareness of hypoglycaemia. Ther Adv Endocrinol Metab. 2021;12:20420188211000248. 33796253 10.1177/20420188211000248 PMC7968015

[14] Gold AE , MacLeod KM , Frier BM . Frequency of severe hypoglycemia in patients with type I diabetes with impaired awareness of hypoglycemia. Diabetes Care. 1994;17:697‐703. 7924780 10.2337/diacare.17.7.697

[15] World Health Organization Rofe . Use of Well‐Being Measures in Primary Health Care—The DepCare Project Health for All. Target 12 E60246. WHO; 1998.

[16] Welch GW , Jacobson AM , Polonsky WH . The problem areas in diabetes scale. An evaluation of its clinical utility. Diabetes Care. 1997;20:760‐766. 9135939 10.2337/diacare.20.5.760

[17] Dulon M , Bardehle D , Blettner M . Assessing social inequality in microcensus data and German national health examination survey. Gesundheitswesen. 2003;65:629‐635. 14639520 10.1055/s-2003-44623

[18] Schumann M . Electronic medical information system for long‐term documentation of chronic diseases (EMIL). 2024.

[19] Seaquist ER , Anderson J , Childs B , et al. Hypoglycemia and diabetes: a report of a workgroup of the American Diabetes Association and the Endocrine Society. J Clin Endocrinol Metab. 2013;98:1845‐1859. 23589524 10.1210/jc.2012-4127

[20] Sommerfield AJ , Ewing FM , Strachan MW , Deary IJ , Aitken G , Frier BM . Self‐treatment of mild symptomatic hypoglycaemia by people with insulin‐treated diabetes. Diabet Med. 2003;20:686‐687. 12873300 10.1046/j.1464-5491.2003.09281.x

[21] Sumner JBC , Williams V . What do patients with type 1 diabetes know about hypoglycaemia? Pract Diab Int. 2000;17:187‐190.

[22] Anderbro T , Amsberg S , Adamson U , et al. Fear of hypoglycaemia in adults with Type 1 diabetes. Diabet Med. 2010;27:1151‐1158. 20854383 10.1111/j.1464-5491.2010.03078.x

[23] Yuksel M , Bektas H . Compliance with treatment and fear of hypoglycaemia in patients with type 2 diabetes. J Clin Nurs. 2021;30:1773‐1786. 33660356 10.1111/jocn.15736

[24] Charleer S , Mathieu C , Nobels F , et al. Effect of continuous glucose monitoring on glycemic control, acute admissions, and quality of life: a real‐World study. J Clin Endocrinol Metab. 2018;103:1224‐1232. 29342264 10.1210/jc.2017-02498

[25] Heinemann L , Freckmann G , Ehrmann D , et al. Real‐time continuous glucose monitoring in adults with type 1 diabetes and impaired hypoglycaemia awareness or severe hypoglycaemia treated with multiple daily insulin injections (HypoDE): a multicentre, randomised controlled trial. Lancet. 2018;391:1367‐1377. 29459019 10.1016/S0140-6736(18)30297-6

[26] Lin R , Brown F , James S , Jones J , Ekinci E . Continuous glucose monitoring: a review of the evidence in type 1 and 2 diabetes mellitus. Diabet Med. 2021;38:e14528. 33496979 10.1111/dme.14528

[27] Mellor J , Kuznetsov D , Heller S , et al. Risk factors and prediction of hypoglycaemia using the Hypo‐RESOLVE cohort: a secondary analysis of pooled data from insulin clinical trials. Diabetologia. 2024;67:1588‐1601. 38795153 10.1007/s00125-024-06177-6 PMC11343909

[28] Busch M , Lehmann T , Wolf G , Gunster C , Müller UA , Müller N . Antidiabetic therapy and rate of severe hypoglycaemia in patients with type 2 diabetes and chronic kidney disease of different stages—a follow‐up analysis of health insurance data from Germany. Exp Clin Endocrinol Diabetes. 2021;11:821‐830. 10.1055/a-1129-6699 32289830

[29] American Diabetes Association Professional Practice C . 6. Glycemic goals and hypoglycemia: standards of care in Diabetes‐2024. Diabetes Care. 2024;47:S111‐S125. 38078586 10.2337/dc24-S006 PMC10725808

[30] Lawton J , Rankin D , Cooke DD , et al. Self‐treating hypoglycaemia: a longitudinal qualitative investigation of the experiences and views of people with type 1 diabetes. Diabet Med. 2013;30:209‐215. 22946549 10.1111/dme.12007

